# Deep Learning-Based Aortic Diameter Measurement in Traumatic Hemorrhage Using Shallow Attention Network: A Path Forward

**DOI:** 10.3390/diagnostics15111312

**Published:** 2025-05-23

**Authors:** Yoonjung Heo, Go-Eun Lee, Jungchan Cho, Sang-Il Choi

**Affiliations:** 1 Division of Trauma Surgery, Department of Surgery, Dankook University College of Medicine, Cheonan-si 31116, Republic of Korea; serapinaoliv@gmail.com; 2 Department of Trauma Surgery, Trauma Center, Dankook University Hospital, Cheonan-si 31116, Republic of Korea; 3 Department of Computer Science and Engineering, Dankook University, Yongin-si 16890, Republic of Korea; ge971010@naver.com; 4 Department of Computing, Gachon University, Seongnam-si 13120, Republic of Korea

**Keywords:** aorta, trauma, hemorrhage, computed tomography, deep learning, image segmentation

## Abstract

**Background/Objectives:** The accurate assessment of aortic diameter (AoD) is essential in managing patients with traumatic hemorrhage, particularly during interventions such as resuscitative endovascular balloon occlusion of the aorta (REBOA). Manual AoD measurements are time-consuming and subject to inter-observer variability. This study aimed to develop and validate a deep learning (DL) model for automated AoD measurement in trauma patients requiring massive transfusion. **Methods:** Abdominal CT scans from 300 adult patients were retrospectively analyzed. A Shallow Attention Network was trained on 444 manually annotated axial CT images to segment the aorta and measure its diameter. An ellipse-based calibration method was employed for enhanced measurement accuracy. **Results:** The model achieved a mean Dice coefficient of 0.865 and an intersection over union of 0.9988. After calibration, the mean discrepancy between predicted and ground truth diameters was 2.11 mm. The median diaphragmatic AoD was 22.59 mm (interquartile range: 20.18–24.74 mm). **Conclusions:** The proposed DL model with ellipse-based calibration demonstrated robust performance in automated AoD measurement and may facilitate timely planning of aortic interventions in trauma care.

## 1. Introduction

Major hemorrhagic trauma and subsequent resuscitation can have varied effects on aortic dimensions. In hypovolemic shock, the aortic diameter (AoD) significantly decreases [1,2,3,4]. Animal studies have demonstrated a strong correlation between the extent of blood loss and reduction in AoD. For instance, the descending thoracic AoD decreased by 8.1% with a 10% blood loss and by 38.2% with a 40% blood loss [4]. Two primary mechanisms may account for this reduction: (1) decreased intraluminal pressure due to diminished circulating blood volume, leading to a collapse of the aortic wall, and (2) vasoconstriction of the aortic wall triggered by the endogenous release of vasoconstrictors during hypovolemia. Furthermore, it is essential to consider that the initially reduced AoD may increase during fluid resuscitation or massive transfusion.

Variability in AoD presents a significant challenge for emergency interventions in trauma patients, particularly in procedures such as thoracic endovascular aortic repair (TEVAR) and the resuscitative endovascular balloon occlusion of the aorta (REBOA). TEVAR aims to reconstruct the aortic wall in cases of blunt thoracic aortic injury, using an endograft tailored to the vessel dimensions visible on computed tomography (CT) imaging. However, in patients with reduced AoD, undersized stents may be selected, increasing the risk of complications such as endoleak and endograft migration [2,4,5]. One study reported that in cases involving a 20% blood loss, the endograft diameter would need to be approximately 42% larger than the initial measurement to accommodate the restored vessel size [4]. REBOA is another lifesaving intervention that temporarily occludes aortic inflow using an inflatable balloon to restore perfusion. Accurate balloon inflation relative to the reduced AoD is essential to avoid complications. However, the optimal saline volume required for inflation depends on the resistance encountered by the balloon as it expands against the vessel wall [6]. Overinflation can increase the risk of catastrophic aortic rupture [7].

Rapid and accurate AoD assessment in emergency settings remains challenging due to the limitations of conventional methods. Clinicians must manually interpret CT scans—a time-intensive process prone to intra- and inter-observer variability and susceptible to diagnostic errors [8]. These issues are exacerbated in high-stress trauma care environments. However, recent advances in artificial intelligence (AI) have shown promise in overcoming these limitations. Deep learning (DL), a subfield of AI focused on building data-driven models, has demonstrated considerable potential in automating and enhancing the analysis of medical images [9].

Recent advances in artificial intelligence have led to the development of deep learning (DL) models for aortic segmentation and diameter measurement across various clinical scenarios. Early approaches utilized convolutional neural networks (CNNs), including fully convolutional networks (FCNs) for region-of-interest (ROI)-based detection of aortic thrombus [10], and hybrid dual-view CNNs for analyzing abdominal aortic aneurysms [11]. Further improvements in diagnostic precision for both contrast-enhanced and non-contrast CT scans have been achieved through multitask learning and attention-augmented CNNs [12,13,14]. Transformer-based architectures, such as the Mixed Transformer U-Net [15] and CNN–Swin Transformer hybrids such as CIS-UNet [16], have enabled more accurate multi-class segmentation of the aorta and its branches by capturing long-range contextual dependencies in addition to Geometry-aware models, such as deep distance transform (DDT) [17]. In parallel, landmark-based segmentation pipelines have been introduced for applications such as thoracic endovascular aortic repair and transcatheter aortic valve implantation, providing accurate localization of the aortic root and valve leaflets [18,19]. Finally, trauma-specific and non-imaging-based approaches have emerged; for example, DeepLabV3+-based body surface segmentation has been used to estimate internal REBOA zones using only external torso topology [20]. These studies highlight the clinical need for efficient, reproducible, and anatomically informed DL frameworks for aortic analysis.

Despite these advancements, studies addressing the dynamic vascular morphology and hypovolemic conditions characteristic of trauma care are limited. The proposed model bridges this gap by providing efficient and calibrated AoD measurements in trauma patients experiencing massive hemorrhage. Therefore, this preliminary study aims to develop and validate a DL model capable of automatically detecting and measuring thoracic AoD in patients with traumatic hemorrhage. Additionally, we implemented an ellipse-based calibration method to improve the accuracy of AoD measurements. By validating this novel approach, we aim to facilitate more effective planning of aortic interventions and lay the groundwork for future investigations into AoD variability and predictive modeling.

The contributions of our research are summarized as follows:

- The proposed DL-based aortic segmentation model automatically measures thoracic AoD from CT images. Unlike those in previous studies, our model focuses on patients with traumatic hemorrhages and enables efficient tracking of clinically significant changes in AoD.
- The model incorporates an attention mechanism to highlight the structural features of the aorta while suppressing irrelevant background information, thereby improving segmentation accuracy. The proposed AoD measurement model, which is based on a 2D DL architecture, is computationally efficient and clinically applicable, enabling rapid and accurate assessment in time-sensitive settings.
- The proposed ellipse-based calibration technique is effective for the segmentation output, which is particularly beneficial given the urgency and procedural requirements of REBOA in critically injured patients. This ellipse-based rejection strategy resulted in a noticeable improvement in overall segmentation performance. Notably, cases flagged by the rejection process can be referred for expert review, thereby enhancing clinical safety.
- When applied to thoracic CT images of 300 patients from a single level I trauma center, the model demonstrated excellent performance. The automatically measured AoDs differed from those obtained by experienced clinicians by approximately 2.11 mm.

## 2. Methods

### 2.1. Study Design and Population

This retrospective study analyzed adult patients treated for traumatic hemorrhage requiring massive transfusion at a level I trauma center. Massive transfusion was defined as the administration of ≥5 units of red blood cells within 4 h or ≥10 units within 24 h [21]. The trauma registry, covering the period from July 2017 to November 2022, initially included 13,517 patients.

Patients with stable hemodynamics following blood or crystalloid resuscitation underwent immediate CT scanning, while those requiring urgent surgical interventions were scanned postoperatively. Three-phase CT imaging (precontrast, arterial, and delayed phases) was performed using an Ingenuity Elite CT scanner (Philips Medical Systems, Cleveland, OH, USA).

**Figure 1:**
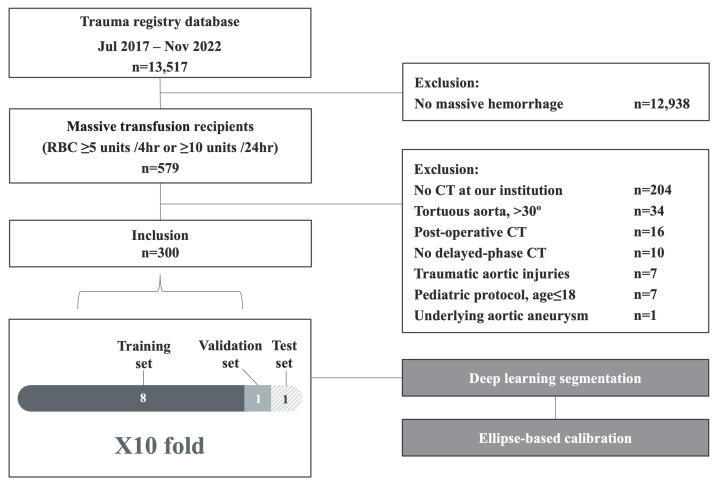
Flowchart showing the patient selection process.

The exclusion criteria included non-massive hemorrhage, CT scans not performed at our institution, severely tortuous aortas (defined as curvature > 30°), traumatic aortic injuries, underlying aortic aneurysms, postoperative CT scans, CTs not acquired with 5 mm slice thickness, absence of delayed-phase images, and pediatric patients (age ≤ 18). The final study cohort comprised 300 patients. The patient selection process is illustrated in Figure 1.

### 2.2. Image Acquisition and Preprocessing

Axial images of the thoracic aorta, extending from the proximal descending segment to the diaphragmatic level, were collected. All images were anonymized and stored in the Digital Imaging and Communications in Medicine (DICOM) format. The 3D DICOM data were converted into 2D image slices to facilitate training of the 2D segmentation network.

We selectively included axial slices covering the thoracic aorta from the proximal descending segment to the diaphragm, as this region is highly relevant for trauma-related interventions, particularly Zone I REBOA placement. A total of 444 images were manually annotated across the cohort to train the DL model. The number of slices per patient varied based on anatomical differences and scan coverage, but was deliberately constrained to ensure consistent labeling and clinical relevance.

Annotations were performed in accordance with the American Heart Association guidelines using the Computer Vision Annotation Tool (CVAT, https://www.cvat.ai/, accessed on 1 March 2024.) for AoD measurement on CT scans [22]. During preprocessing, image intensities were normalized based on the mean and standard deviation of the pixel intensity values. CT Hounsfield units were converted to the red–green–blue (RGB) color space for visualization purposes. Data augmentation included random horizontal and vertical flips and rotations, each applied with a 50% probability. All images were resized to 352 × 352 pixels. Segmentation labels were created by marking the regions corresponding to the aorta. Given the cylindrical structure of the aorta, its cross-section generally appears circular; accordingly, we applied circular annotations to reflect this anatomical characteristic as accurately as possible.

### 2.3. DL Model Development and Training

**Figure 2:**
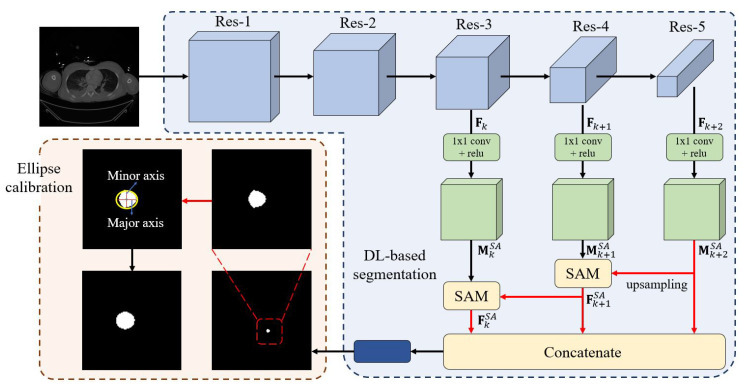
Overview of the aortic diameter measurement pipeline. The pipeline included deep learning-based segmentation for aortic detection and ellipse-based calibration for accurate diameter measurement. The input CT image was processed through residual blocks (Res-1 to Res-5) to extract multi-scale features. Feature maps from each block were passed through a 1 × 1 convolution layer followed by batch normalization and ReLU activation. The Res-5 output was upsampled and concatenated with shallower features. The resulting feature map was then processed through a final 1 × 1 convolution layer to generate the segmentation mask. During training, a sigmoid activation function produced an initial binary mask. The major and minor axes were then computed from the polygonal segmentation, which was transformed into an elliptical boundary using ellipse-based calibration.

We developed an automated pipeline integrating a DL network with ellipse-based calibration to segment the aorta and measure AoD in CT images. The network architecture is flexible; in this study, we implemented a Shallow Attention Network (SANet) [23] with a Res2Net [24] backbone (see Figure 2).

Let $F_{k}\in R^{L\times M\times N}$ represent the *k*-th feature map in Figure 2, where *L*, *M*, and *N* denote the height, width, and number of channels, respectively. When $F_{k}$ contains only a single high-level semantic feature, the feature map $M_{k}^{SA}$ and the corresponding shallow attention-enhanced feature map $F_{k}^{SA}$ from the Shallow Attention Module (SAM) can be expressed as

(1) $$\begin{array}{c} M_{k}^{SA}=r\left(Conv\left(F_{k}\right)\right),\;\;F_{k}^{SA}=up\left(F_{k+1}^{SA}\right)⊗M_{k+1}^{SA}, \end{array}$$

where r(·) denotes the ReLU activation function, Conv(·) denotes a 1×1 convolutional layer, and ⊗ represents element-wise multiplication. The upsampling function up(·) aligns the deeper-level feature map $F_{k+1}^{SA}$ with that of the shallower-level feature map $M_{k}^{SA}$. Furthermore, if a deeper feature map $F_{k+2}$ is available, representing a higher-level semantic abstraction than $F_{k+1}$ within the *k*-th stage, it is incorporated to enhance the shallow attention by providing additional contextual information. In this case, the updated shallow attention map $M_{k+1}^{SA}$ is accordingly computed.

(2) $$\begin{array}{c} M_{k+1}^{SA}=r\left(Conv\left(F_{k+1}\right)\right),\;\;F_{k+1}^{SA}=up\left(M_{k+2}^{SA}\right)⊗M_{k+1}^{SA} \end{array}$$

All experiments were conducted on a Linux 22.04 and country from where the equipment was sourced. please check and modify full text server equipped with an Intel(R) Core(TM) i7-13700 CPU @ 3.00 GHz, 128 GB RAM, and an NVIDIA GeForce RTX 4090 GPU (24 GB VRAM). Model training used PyTorch 1.8.0+cu118, Python 3.7, and CUDA 11.8. The model was trained for 100 epochs (approximately 34 s per epoch) using the Adam optimizer, with a learning rate of 0.4, batch size of 16, and input resolution of 352 × 352.

The dataset was divided into training, validation, and test sets in an 8:1:1 ratio. Ten-fold cross-validation was applied. For Fold 1, subjects 1–30 were used for testing, with the remainder for training and validation. This procedure was repeated for subsequent folds (e.g., subjects 31–60 for Fold 2 testing), ensuring that the entire dataset was eventually used for both training and evaluation.

During the training process, we observed the accuracy and precision trends across epochs and confirmed that the model successfully converged to a global optimum.

### 2.4. Ellipse Fitting of the Aorta

Because the aorta often does not run perpendicular to the CT scan plane, direct diameter measurements may be inaccurate. To address this, we applied an ellipse-fitting method using OpenCV to convert the segmented polygonal aortic boundary into an ellipse (Figure 2). First, the polygon’s contour was extracted using the findContours function, followed by fitEllipse to compute the major and minor axes, center coordinates, and angle of inclination.

These values were used to reconstruct the fitted ellipse based on the standard equation:

$$\left(x-x_{0}\right)/a^{2}+\left(y-y_{0}\right)/b^{2}=1,$$

where *a*, *b*, $x_{0}$, and $y_{0}$ represent the axes of the ellipse and center coordinates, respectively.

We defined the minor axis of the fitted ellipse as the AoD (calibrated diameter), as it provides more clinically relevant information than the major axis in the context of the REBOA procedure.

To calculate the real-world AoD, we applied the DICOM tag PixelSpacing, which denotes the physical distance per pixel. The scaling ratio between the original and resized (352-pixel) images was calculated and used to convert the measured pixel value into millimeters. Discrepancies between predicted and ground truth AoD values were used to assess model error.

### 2.5. Model Evaluation

The performance of the segmentation model was evaluated using the Dice coefficient and intersection over union (IoU) metrics. For each image, a confidence score between the ground truth and predicted segmentation was calculated. The overall model performance was reported as the average across all test datasets. Since 10-fold cross-validation was used, the final performance represents the mean of the evaluation metrics across all 10 folds.

## 3. Results

### 3.1. Patient Characteristics

Table 1 presents the demographic and clinical characteristics of the study cohort. The majority of patients (80.0%) were male, with a mean age of 53.6 years. Most patients (96.3%) sustained blunt trauma. The median Injury Severity Score was 30.0 (interquartile range: 25.0–38.0). The mean height, body weight, and body mass index were 168.5 cm, 67.4 kg, and 23.6 kg/m^2^, respectively.

### 3.2. DL Segmentation

The DL model was applied to 4277 abdominal CT images obtained from 300 patients who underwent massive transfusion for traumatic hemorrhage. Using augmentation techniques such as random flipping and rotation, we generated a total of 8544 images to train the deep neural network. Due to anatomical variation in thoracic aorta length, the model processed between 5 and 25 axial CT slices per subject to segment the aorta and compute the corresponding AoDs.

**Figure 3:**
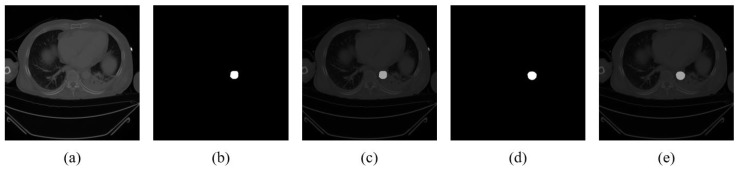
Segmentation results on aortic CT images. (**a**) Original CT image. (**b**) Ground truth segmentation mask. (**c**) Overlay of original image and ground truth. (**d**) Predicted segmentation mask. (**e**) Overlay of original image and predicted mask.

Minimal visual discrepancy between the ground truth and predicted segmentation masks was observed in the overlay images, highlighting the effectiveness of the SANet segmentation model (Figure 3). Dice and IoU scores were computed for each fold, with overall averages across all folds calculated. The model achieved a mean Dice coefficient of 0.865 and a mean IoU of 0.9988 (see “w/o rejection’’ column in Table 2).

To ensure the validity of AoD measurements, we assumed the presence of a single aorta per image. Cases in which multiple ellipses were detected or ellipse fitting failed were excluded. This led to the rejection of approximately 8.7% of test images, resulting in successful AoD measurement in 3905 of 4277 images. The “w/ rejection’’ columns in Table 2 show the Dice and IoU values for images in which ellipse fitting was successful. These metrics improved following the rejection of suboptimal segmentations, underscoring the benefit of our quality control strategy.

We further evaluated the generalizability of the ellipsoid calibration method by replacing the backbone network with U-Net [25]. When using U-Net, the rejection strategy excluded approximately 11.2% of the test images. As shown in Table 3, ellipsoid fitting-based rejection consistently improved performance, even when applied to the U-Net architecture. Moreover, a comparison between Table 2 and Table 3 demonstrates that SANet outperforms U-Net across all evaluation metrics. This performance gain can be attributed to integrating a SAM into the encoder–decoder architecture of U-Net, which enhances the ability of the model to capture aortic features. In summary, combining SANet with the proposed ellipsoid-based method results in significant performance improvements over U-Net while maintaining computational efficiency.

### 3.3. Performance of AoD Measurement

**Figure 4:**
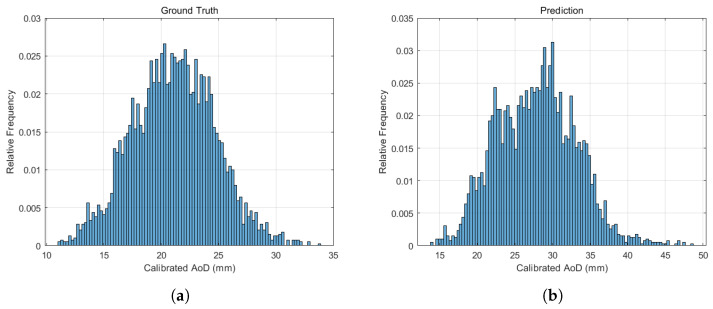
Relative frequency distributions of (**a**) calibrated AoD values based on manual clinician annotations and (**b**) AoD values automatically measured by the model. Both distributions exhibit a Gaussian-like shape.

We assessed discrepancies between calibrated AoDs derived from clinician-annotated segmentation masks (ground truth) and those obtained from the model-generated masks. Figure 4 shows the relative frequency distributions of AoD measurements from both sources. The distribution of manual measurements (Figure 4a) followed a Gaussian pattern, indicating sufficient sample size and measurement reliability. The automatically measured AoDs (Figure 4b) also exhibited a Gaussian-like distribution, suggesting that the model captured physiological variation effectively. Detailed results are presented in Supplementary Material S1. The mean absolute error between predicted and ground truth AoD values was 2.11 mm, based on 3905 successfully measured images. These findings confirm the model’s high accuracy and reliability in quantifying thoracic AoD.

AoD measurements at the diaphragmatic level were unavailable for 16 of the 300 patients. Among those with available data, the median diaphragmatic AoD was 22.59 mm, with an interquartile range of 20.18–24.74 mm (see Supplementary Material S2).

## 4. Discussion

In trauma care, the prompt recognition of a patient’s condition and timely decision making are critical for improving survival outcomes [26]. The management of severe trauma often requires a labor-intensive and multidisciplinary approach [27]. Inter- and intra-observer variability remain inherent challenges when radiologists are required to manually measure AoD on CT images [28]. Even among experienced radiologists, inter-observer variability tends to be greater in the transverse plane, and both inter- and intra-observer differences increase with larger vessel diameters [28]. Recent studies have shown that AI-assisted tools can reduce such variability and enhance efficiency. In Rueckel et al.’s work [29], AI software achieved measurement errors comparable to manual inter-reader variability, maintaining consistent performance even in low-dose chest CT protocols. These findings underscore the potential of AI to deliver faster, more reproducible, and standardized aortic measurements. Nonetheless, the clinical adoption of DL remains limited by challenges such as model overfitting, inadequate evaluation strategies, and the use of non-generalizable datasets [9,30].

DL-based aortic segmentation using CT imaging has evolved substantially over the past decade. Table 4 summarizes recently proposed models categorized by architectural design, including CNNs, transformer-enhanced frameworks, geometry-aware approaches, and trauma-specific solutions. Most of these models were developed for hemodynamically stable and non-emergent cases [10,11,12,17,18]. Others introduced architectural innovations—such as transformer-based encoders [15,16], multitask learning frameworks [13], and geometric refinement techniques [31]—to improve segmentation performance in complex vascular anatomies.

While our study shares similarities with these previous works in terms of aortic segmentation and AoD measurement, it differs in several key aspects. First, our work focuses specifically on trauma patients with hypovolemic shock—a population not addressed in previous studies. Second, our model is designed to accommodate the dramatic changes in AoD associated with hemorrhagic shock. Third, we introduce an ellipse-based calibration method to enhance measurement precision, which is particularly relevant in trauma scenarios requiring endovascular interventions.

**Figure 5:**
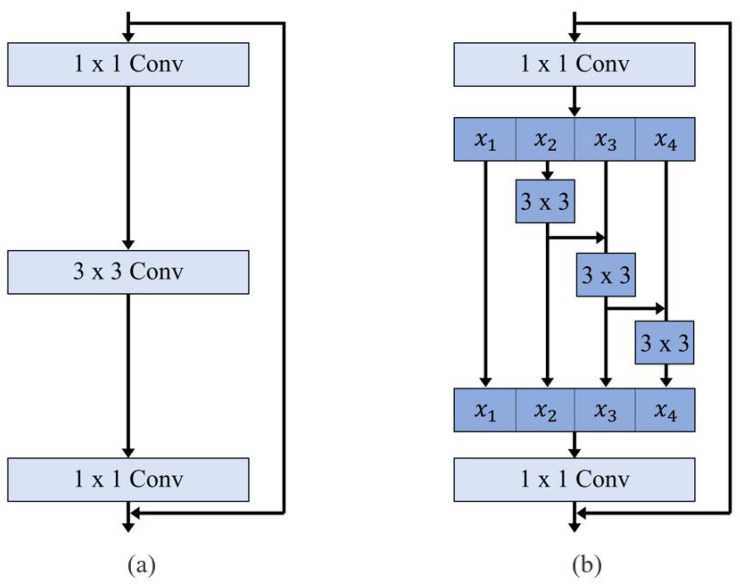
Comparison between (**a**) ResNet block and (**b**) Res2Net block. The Res2Net block replaces traditional 3 × 3 convolution layers with a hierarchical residual-like structure to capture multi-scale features at a finer granularity.

Although our study does not introduce an entirely new pipeline, it highlights the utility of an openly accessible SANet architecture. Originally proposed for polyp detection in colorectal cancer screening, SANet employs a Shallow Attention Module to enhance focus on the target structure while minimizing background interference [23]. This improves segmentation accuracy, particularly in grayscale CT images. Additionally, SANet uses Res2Net as its backbone, which captures multi-scale features through a hierarchical residual-like structure, expanding the receptive field and enhancing contextual learning (Figure 5) [24]. By implementing an accessible and reproducible framework, we enable other researchers and clinicians to adopt and validate our method, promoting broader clinical application. The small mean discrepancy (2.11 mm) between predicted and ground truth AoD measurements further supports the effectiveness of our ellipse-based calibration method.

Several limitations must be acknowledged. The retrospective, single-center design introduces potential selection bias. Technical factors, including variability in contrast enhancement, patient hemodynamics, and scanning techniques, may have influenced model performance. The absence of ECG gating introduces possible inaccuracies due to motion artifacts. In addition, variability in CT cropping protocols across technologists led to differences in the length of aorta analyzed. Diameter estimation may be affected by the exclusive use of axial slices, especially in patients with aortic tortuosity. Our geometric calibration approach, while effective, may be insufficient in cases involving focal aortic pathologies. Ground truth labels derived from manual annotations are inherently prone to inter-observer variability, which could influence model training and validation. Future implementation of 3D centerline analysis is warranted, as prior studies suggest it improves both segmentation accuracy and inference time compared to 2D approaches [33]. The 8.7% failure rate in AoD measurements emphasizes the need for human oversight. In cases where segmentation quality is inadequate, clinician review remains essential. Therefore, our DL model should be considered a supportive tool, not a replacement for human expertise [30,34].

Despite these limitations, our study underscores the feasibility and clinical utility of DL-based automated AoD measurement, particularly at the diaphragmatic level. This level serves as a reliable anatomical landmark, facilitating consistent measurement across patients and imaging protocols [22]. Additionally, periaortic hematomas near the diaphragm may signal associated injuries, including aortic rupture, diaphragmatic tears, or vertebral fractures [35]. In REBOA procedures, the occlusion balloon is typically deployed just above the diaphragm [6]. Thus, accurate AoD assessment at this level is crucial for safe and effective intervention.

While we report a median diaphragmatic AoD, it is important to note that this measurement is influenced by various factors, including age, height, weight, and blood pressure. Accordingly, future research will focus on analyzing the effects of these clinical and demographic variables on AoD in trauma patients. Our long-term objective is to develop AI-based predictive models that can estimate AoD using patient-specific parameters. Such tools could assist in determining the optimal balloon inflation volume, improving resuscitation outcomes while minimizing the risk of aortic injury.

In conclusion, we designed a DL model for automated AoD measurement in patients with traumatic hemorrhage. Based on the SANet architecture, our model demonstrated strong performance in segmenting the aorta and accurately measuring its diameter from enhanced CT images. By automating this process, the model may facilitate timely and effective aortic interventions. Future work should focus on clinical translation, multicenter validation, and adaptation to diverse patient populations and imaging settings.

## Figures and Tables

**Table 1:** Patient characteristics, initial hemodynamics, and laboratory results.

Variables	Overall (n = 300)
Age (y)	53.6 ± 17.7
Sex, male (%)	240 (80.0)
Body mass index (km/m^2^)	23.6 ± 3.3
Height (cm)	168.5 ± 7.8
Body weight (kg)	67.4 ± 11.9
Injury mechanism, blunt (%)	289 (96.3)
Injury severity score *	30.0 (25.0, 38.0)
Abbreviated trauma score	
1. Head	2.6 ± 2.2
2. Chest	2.4 ± 1.6
3. Abdomen	1.7 ± 1.6
4. Extremities	2.0 ± 1.7
Initial vital signs *	
1. Systolic blood pressure (mmHg)	103.5 (70.0, 135.0)
2. Heart rate (beats/min)	100.0 (74.8, 118.0)
3. Revised trauma score	9.0 (7.0, 10.0)
Initial laboratories *	
1. pH	7.3 (7.2, 7.4)
2. Base excess (mmol/L)	−5.8 (−10.4, −2.0)
3. Lactate (mmol/L)	4.8 (3.0, 8.1)
4. Hemoglobin (g/dL)	11.9 (10.2, 13.2)
In-hospital mortality (%)	149 (49.7)

Data are presented as n (percentage) or mean ± SD. * Median value (interquartile range).

**Table 2:** SANet performance assessed using Dice coefficients and intersection over union (IoU).

Test Set	Dice		IoU	
	**w/o Rejection**	**w/ Rejection**	**w/o Rejection**	**w/ Rejection**
Fold 1	0.8776	0.9025	0.9987	0.9991
Fold 2	0.8548	0.8891	0.9988	0.9990
Fold 3	0.8728	0.9102	0.9988	0.9990
Fold 4	0.8211	0.8483	0.9984	0.9988
Fold 5	0.8693	0.8872	0.9990	0.9994
Fold 6	0.8701	0.9004	0.9987	0.9991
Fold 7	0.8978	0.9122	0.9991	0.9993
Fold 8	0.8705	0.8997	0.9987	0.9990
Fold 9	0.8480	0.8739	0.9988	0.9992
Fold 10	0.8682	0.8943	0.9987	0.9991
Fold average	0.8650	0.8918	0.9988	0.9991

**Table 3:** U-Net performance assessed using Dice coefficients and intersection over union (IoU).

Test Set	Dice		IoU	
	**w/o Rejection**	**w/ Rejection**	**w/o Rejection**	**w/ Rejection**
Fold 1	0.7824	0.8145	0.9211	0.9345
Fold 2	0.7591	0.7733	0.8935	0.9077
Fold 3	0.7763	0.7992	0.9048	0.9217
Fold 4	0.7199	0.7345	0.8887	0.9066
Fold 5	0.7549	0.7861	0.8994	0.9091
Fold 6	0.7688	0.7819	0.9042	0.9244
Fold 7	0.7894	0.8037	0.9178	0.9317
Fold 8	0.7666	0.7911	0.8944	0.9112
Fold 9	0.7331	0.7658	0.8773	0.8947
Fold 10	0.7543	0.7883	0.9013	0.9176
Fold average	0.7604	0.7838	0.9002	0.9159

**Table 4:** Comparison of deep learning models for aortic segmentation using CT scans, grouped by architecture type. “Unchecked AoD” indicates that the study reports segmentation results but does not provide diameter measurements.

Study	Year	Method	Input	AoD	Features
**2D/3D CNN-Based Models**					
López-Linares et al. [10]	2018	FCN + ROI Detection	CTA	✓	Fully automatic thrombus segmentation using fully convolutional network (FCN) and region of interest (ROI) modeling in post-EVAR CTA.
Chandrashekar et al. [12]	2022	Attention U-Net	CTA + NCCT	✓	Uses attention modules for thrombus and lumen segmentation across both contrast and non-contrast CTs (NCCT).
Brutti et al. [11]	2022	Dual-view CNN	CTA	✓	Employs dual-view CNN from axial and coronal planes to assess thrombus and aortic wall.
Yang et al. [13]	2023	Multitask CNN	NCCT	✓	Simultaneous aortic segmentation and anatomical landmark localization on NCCT with a squeeze-excitation CNN.
Lo Piccolo et al. [14]	2023	Retrained 3D U-Net	Chest CT	✓	Improved robustness for AoD measurement in non-ECG-gated CT by retraining on local clinical data.
**Transformer-Based and Hybrid CNN Models**					
Wang et al. [15]	2021	Mixed Transformer U-Net	CT	✓	Combines CNN encoding with global–local self-attention and external memory for enhanced vessel context learning.
Imran et al. [16]	2024	CIS-UNet (CNN + Swin Transformer)	CTA	✓	Performs multi-class segmentation of aorta and 13 branches with transformer-enhanced CNN encoder.
**Geometry-Aware Models**					
Wang et al. [31]	2020	Deep Distance Transform (DDT)	CT	✓	Predicts distance maps and tubular cross-sectional radii for geometry-aware segmentation and scale estimation.
**Landmark-Based TAVI and Diameter Pipelines**					
Lalys et al. [18]	2019	Statistical + Deformable Hybrid	ECG-gated CTA	✓	Aortic root and annulus landmark detection using statistical atlases and active contours; designed for TAVI planning.
Pradella et al. [19]	2021	CNN + Landmark Detection	ECG-gated CTA	✓	Fully automated, guideline-compliant AoD measurement with DL-based centerline fitting and landmark detection.
**Dissection and Triage-Specific Models**					
Harris et al. [32]	2019	CNN Classifier	CTA		Dissection and rupture detection via CNN-based emergency triage tool in post-contrast CT.
Xiang et al. [17]	2023	ADSeg (Flap Attention)	CTA	✓	Specialized attention for intimal flap delineation in aortic dissection cases using ADSeg.
**Trauma and REBOA-Specific Models**					
Takata et al. [20]	2023	DeepLabV3+	External Body Surface (2D)		Predicts internal REBOA zones by segmenting external torso surfaces; requires no aortic imaging.

## Data Availability

The original contributions presented in this study are included in this article as supplementary materials; further inquiries cans be directed to the corresponding authors.

## Supplementary Materials

The following supporting information can be downloaded at https://www.mdpi.com/article/10.3390/diagnostics15111312/s1, Table S1: The full set of thoracic aortic diameters (AoD) calculated from x and y coordinates and PixelSpacing values through deep learning based segmentation; Table S2: The aortic diameter values measured at the diaphragm level for each patient.
